# How do appraisal as threat or challenge, efficacy, and environmental quality affect wellbeing in the COVID-19 pandemic?

**DOI:** 10.3389/fpsyt.2022.1009977

**Published:** 2023-01-04

**Authors:** Hannah Wallis, Veronique Holzen, Theresa Sieverding, Ellen Matthies, Karolin Schmidt

**Affiliations:** ^1^Medical Faculty, University Clinic of Psychosomatic Medicine and Psychotherapy, University Hospital Magdeburg, Otto-von-Guericke-University Magdeburg, Magdeburg, Germany; ^2^Department of Environmental Psychology, Faculty of Natural Sciences, Institute of Psychology, Otto-von-Guericke-University Magdeburg, Magdeburg, Germany

**Keywords:** subjective wellbeing, threat appraisal, environmental quality, efficacy, COVID-19

## Abstract

**Background:**

In crises, it is of great relevance to identify mechanisms that help people to maintain a certain level of wellbeing. This paper investigates whether appraising the COVID-19 pandemic as a threat vs. as a challenge has different effects on subjective wellbeing during the pandemic. Furthermore, we study the role of the perceived local environmental quality for individuals' subjective wellbeing.

**Methods:**

*Via* online survey study with two times of measurement (*N* = 758), we investigated (a) the prediction of participants' wellbeing in June 2020 and June 2021 through five variables and (b) how these five variables moderated within-participant differences in subjective wellbeing over time.

**Results:**

Results showed that a stronger perception of the pandemic as a threat (feeling worried) and a lower education in June 2020 predicted a lower subjective wellbeing in 2020 and 2021. A stronger challenge appraisal (feeling confident), higher efficacy expectations, and positive perceptions of the local environmental quality in June 2020 predicted a higher wellbeing in 2020 and 2021. There was no substantial change in participants' aggregated wellbeing over time. However, those who perceived the pandemic more as a threat in June 2020 struggled more with negative changes in their wellbeing, whereas those who perceived the pandemic more as a challenge reported a higher wellbeing.

**Conclusion:**

It seems key to support people in activating positive feelings to successfully cope with crises.

## Introduction

Since the beginning of the COVID-19 pandemic in the spring of 2020, there have been many discussions about what could help people maintain a certain quality of life despite the threat of the pandemic (1). The COVID-19 pandemic severely affected people's daily lives, for example through contact regulations, the closure of restaurants, or the cancellation of cultural events. Feelings of being vulnerable and fearing for one's health or the health of others negatively affected people's subjective wellbeing and mental health. Accordingly, the prevalence of elevated depressive symptoms increased from 27.8% in 2020 to 32.8% in 2021 (2). Studies suggest that, during the pandemic, people experienced a stronger sense of threat to their physical and psychological health (3).

Our goal was hence to identify psychological and environmental factors that allow people to cope with crises such as the current pandemic (4). We took a two-fold perspective, examining coping styles that might enable people to maintain their subjective wellbeing during the COVID-19 pandemic and perceptions of the local environment that might foster people's subjective wellbeing. Our aim was to investigate whether participants' wellbeing in June 2020 and June 2021 could be predicted through the five (between) variables measured in June 2020 (threat appraisal, challenge appraisal, efficacy expectations, local environmental quality, and education).

Several studies applied measures of subjective wellbeing to investigate how the overall quality of life was affected by pandemic-related changes. Survey data monitoring life satisfaction in Germany (5) shows various ups and downs of subjective life satisfaction (as a proxy for subjective wellbeing) that can be associated with changes in infection rates. Other studies have examined psychological and physical predictors of subjective wellbeing in different demographic groups such as students (6), medical staff (7), or families (8). Results indicate that, e.g., the fear of oneself (9) or family members getting infected, dealing with financial insecurity, academic or work related stress, and parental stress had a negative impact on subjective wellbeing. Therefore, it seems crucial to gain a better understanding of psychological and environmental factors that help maintain a certain level of subjective wellbeing to antagonize these negative effects during the pandemic. For example, a study from March 2020 from Germany indicated that higher subjective wellbeing was related to active coping, hence people appraising the pandemic as challenge and controllable-by-self (10). In line with these results, we studied how the emotional appraisal of the pandemic and efficacy expectations predicted subjective wellbeing in a representative sample in Germany during different stages of the pandemic (June 2020 and June 2021). In addition, we investigated whether the perceived local environmental quality could positively affect subjective wellbeing during the pandemic. We drew from a long research tradition showing the positive impact that access to a clean natural environment and a high environmental quality can have on a person's subjective wellbeing and life satisfaction (11–13).

Without question, experiencing a crisis such as the COVID-19 pandemic puts stress on individuals. For years, the Transactional Theory of Stress (TTS) (14, 15) has been used successfully to investigate how individuals experience and cope with stressful situations and how this experience affects their wellbeing. Drawing from this theory, we focused on whether appraising the COVID-19 pandemic as a threat vs. as a challenge would differently affect participants' subjective wellbeing during the pandemic. Some studies already applied the broader idea of TTS to wellbeing in the COVID-19 pandemic (16–18). However, studies using the original constructs of Lazarus and colleagues differentiating between people's perception of the pandemic as threat vs. as challenge are rare. Studying these specific coping mechanisms in a large representative sample contributes to the literatures on stress appraisals in the pandemic. To do so seems necessary in order to develop effective communication strategies to support people who feel helpless and to support effective coping strategies (10) in crises.

### Appraisal of the COVID-19 pandemic as a threat vs. a challenge

Psychological theories on coping with stress suggest that problem appraisal is a relevant predictor of individuals' subjective wellbeing. The TTS (15) postulates that appraising an event as a threat may impair subjective wellbeing whereas perceiving it as a challenge may be linked to a higher subjective wellbeing. Threat appraisal involves the belief that one does not have sufficient personal resources to deal with current events and, therefore, perceives oneself as being in danger of harm or loss. In contrast, challenge appraisal entails the assessment that one has sufficient resources to cope with current events and can achieve personal gains or growth when mobilizing physical and psychological energy (10).

Zacher and Rudolph (10) could show that participants' subjective wellbeing was associated with both stress appraisal and coping strategies in the pandemic. Appraising the pandemic as a threat was related to lower levels of and stronger decreases in subjective wellbeing. Challenge appraisal was positively related to higher wellbeing but did not emerge as a significant predictor for wellbeing in the overall regression model. Supporting the decreasing influence of threat appraisal on individuals' subjective wellbeing, a study from Pakistan found that particularly the fear of getting infected with the virus predicted lower levels of wellbeing since it lead participants to self-isolate more frequently. Simultaneously, the ability to adapt (coping) was related to a higher subjective wellbeing (19).

### Efficacy expectations as a predictor

We studied the potential role of collective efficacy expectations as an additional predictor of subjective wellbeing in the COVID-19 pandemic. This idea is rooted in a long tradition of research studying how people gain or regain agency and feelings of collective efficacy and how these feelings influence subjective wellbeing (20). According to Bandura, agency and feelings of efficacy are of particular relevance in times of crises since they are crucial for being resilient or regaining agency. Results of a longitudinal survey from Germany suggests self-efficacy as a protective factor for life satisfaction and mental health (21). A study by Mækelæ et al. (22) reported that peoples' “thriving” (a construct which has a significant overlap with collective efficacy) reduces their perceived stress in COVID-19 times, which in turn has had a positive effect on their subjective wellbeing.

Drawing from this theoretical and empirical evidence, we expected individuals with higher collective efficacy expectations to be more likely to commit to a mission, be resilient to adversity, and accomplish their goals together with others. Therefore, we expected participants' goal-focused collective efficacy expectations (23) to positively affect their subjective wellbeing.

### Local environmental quality as a buffer for subjective wellbeing

A deeper understanding of environmental factors influencing subjective wellbeing is critical to understand and enhance people's subjective wellbeing in crises in the long run. First studies investigated the positive effect of environmental quality on wellbeing in the COVID-19 pandemic and indicate that access to high quality physical infrastructures and social resources were indeed related to a higher wellbeing (4). Green environments were helpful in enhancing participants' subjective wellbeing (24), particularly in cities (25). There is empirical research showing significant improvements in several environmental quality-dimensions during the pandemic. These studies provide data for diverse countries showing higher air quality in COVID-19 restriction times (26). This higher air quality due to lower (motorized) mobility levels during the pandemic (27) could imply health and wellbeing benefits of spending time in local environments. During the COVID-19 pandemic, people stayed in smaller radiuses from their homes, thus spent more time in local environments. This is supported by evidence from our own data showing higher walking activities of Germans in COVID-19 times [see (28)]. Therefore, it seemed relevant to study the perceived local environment as a potential coping mechanism for subjective wellbeing in the pandemic. We focused on local environments since they were accessible to individuals even during contact and mobility restrictions and when social interactions and support become rare and access to leisure activities more difficult.

### Age, education, income

Besides psychological and environmental factors, past research has shown that sociodemographic variables correlate with subjective wellbeing, in general and during the pandemic. Studies suggest that the subjective wellbeing of people with lower income and education was more negatively affected by the pandemic than it was the case for more advantaged persons, and that the COVID-19 pandemic amplified existing health inequalities (29–31). Lower subjective standards of living and a poorer self-rated health were related to a sharper drop in life satisfaction and a stronger increase in the prevalence of depressive symptoms during the pandemic (32). Moreover, depressive symptoms increased among older adults and adaptive behavior decreased for those with a poorer perceived health (33). These results clearly show the importance of sociodemographic contexts when it comes to subjective wellbeing. In this study, we focused on differences in predictors of wellbeing related to age and investigated, whether a lower education would be related to a lower subjective wellbeing.

## Method

**Figure 1:**
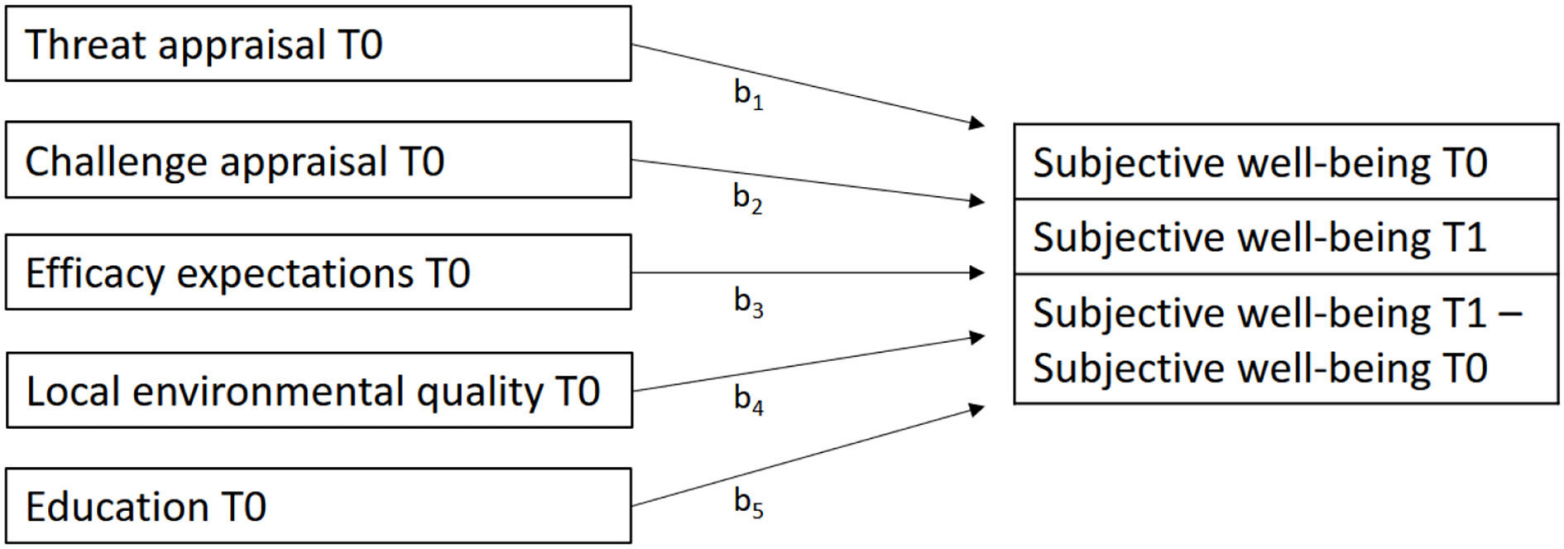
Path diagram representation of the moderation model for the two-occasion within-subjects repeated measure design. Displayed is the model to explain both subjective wellbeing at T0 (June 2020) and T1 (June 2021) as well as the change in subjective wellbeing. b_1_ to b_5_ represent the effects of each variable, respectively, of each moderator on the respective outcome variable.

The SPSS Macro MEMORE (34) was used to analyze the effect of multiple moderators in a two-occasion within-subjects repeated measures design [e.g., (35)]. We tested whether participants' subjective wellbeing in June 2020 and June 2021 could be predicted through the five (between) variables measured in June 2020 (threat appraisal, challenge appraisal, efficacy expectations, local environmental quality, and education). Furthermore, we tested whether the (within) difference in participants' subjective wellbeing measured in June 2020 and in June 2021 could be predicted through these five (between) variables (Figure 1).

### Sample and procedure

The present study was designed and conducted following the APA guidelines on the ethical conduct of research. Data was collected as part of two large online surveys examining the nexus between the COVID-19 pandemic and sustainability [e.g., the influence of the pandemic on sustainable mobility (28); sustainable food consumption (36) and predictors of mitigation measures (37)]. This paper focuses on individuals' subjective wellbeing in the COVID-19 pandemic and related psychological and environmental aspects.

Data collection for the first measurement time (T0) took place from end of June to the beginning of July 2020, after the strictest restrictions related to the so-called first COVID-19 wave were eased in Germany. Participants were recruited and financially compensated by a national panel provider. A total of 3,357 people completed the survey and passed the attention check. Out of those, 265 participants were excluded based on answering time, answers to open format questions, and missing values. *N* = 3,092 people formed the final sample (T0). The sample was roughly representative for the German population with regards to age, gender and for education. The second survey (T1) was conducted in June 2021, just after the third COVID-19 wave hit Germany. Invitations to take part in the survey were sent to the 3,357 participants that took part in the first survey and recruitment ended (due to financial restrictions) with 996 participants who had passed the attention check in the survey. Out of these, 233 were excluded due to answering time (relative speed index *RSI* ≥ 2.0) (38), answers to open format questions, straight lining, and missing codes for assignment to the first wave.

The final sample, including participants that completed both Survey 1 (T0) and Survey 2 (T1), consisted of *N* = 763 participants. Participants' age ranged from 19 to 70 years (*M* = 50.71, *SD* = 12.64), 45.3% were female.

### Measures

Subjective wellbeing (T0 & T1) was measured in line with the widely used short measure of subjective wellbeing, using measures of happiness and life satisfaction as a proxy [see, e.g., (39)]. The items were: “Taking all things together, how happy would you say you are?” and “All things considered, how satisfied are you with your life as a whole nowadays?” (T0: *r* = 0.87, *p* < 0.001; T1: *r* = 0.86, *p* < 0.001). Answers were assessed on a scale ranging from 0 (extremely unhappy/extremely dissatisfied) to 10 (extremely happy/extremely satisfied).

Threat appraisal (T0) was measured in line with Folkman and Lazarus (16). The four items focused on people's anticipatory threat emotions, such as feeling worried, fearful, and anxious. Items were introduced with the following statement: “Please think back to the beginning of the corona crisis.” Participants were then asked to report their agreement with the following statements: “I felt a strong personal threat to my health,” “I was very worried about my relatives,” “I was very worried about what would follow from the crisis worldwide,” and “I am, and I was, very worried” (α = 0.88). Items were measured on a 7-point Likert scale ranging from 1 (strongly disagree) to 7 (strongly agree).

Challenge appraisal (T0) was measured in line with Folkman and Lazarus (16) with four items focusing on anticipatory challenge emotions, such as feeling confident, hopeful, and eager. Again, participants were asked to think back to when the pandemic started. They were asked to indicate their agreement with the following statements: “I was fascinated by the changes we were all capable of,” “I found it exciting to face my habits,” “I saw the crisis as an opportunity for our society,” and “I still see the crisis as an opportunity today” (α = 0.90). Items were again measured on a 7-point Likert scale ranging from 1 (strongly disagree) to 7 (strongly agree).

Efficacy expectations (T0) in the COVID-19 pandemic were measured drawing from studies in the context of the climate crisis (23, 40) with three items: “Together with others who make efforts to contain the corona pandemic, I can make a substantial contribution to contain the corona pandemic,” “I believe that together with others who make efforts to contain the corona pandemic I can contribute to contain the corona pandemic,” and “The joint actions as people who make efforts to mitigate the corona pandemic can make a substantial contribution to mitigate the pandemic” (α = 0.95). Items were again measured on a 7-point Likert scale ranging from 1 (strongly disagree) to 7 (strongly agree).

Local environmental quality (T0) was measured in line with an item from the Umweltbewusstseinsstudie, a recurring survey from the German Environment Agency (UBA) on Germans' environmental awareness. Participants were asked: “Thinking about the last 3 months, how would you rate the quality of the environment in your city, your local community?” Their answer was measured on a 4-point scale ranging from 1 (very good) to 4 (very bad) and recoded for all analyses.

## Results

### Descriptive analyses

A repeated-measures ANOVA was conducted to examine potential differences in subjective wellbeing between June 2020 and June 2021. Participants reported a slightly, but not significantly, higher subjective wellbeing in June 2021 compared to June 2020 [*F*_(1, 751)_ = 3.29, *p* = 0.07; see Table 1].

**Table 1:** Sociodemographic and descriptive characteristics.

	**June 2020–T0**			**June 2021–T1**		
	***N***	***M***	***SD***	***N***	***M***	***SD***
^a^Subjective wellbeing	758	6.77	2.32	756	6.89	2.25
**Moderators**						
^b^Threat appraisal	762	4.35	1.65			
^b^Challenge appraisal	759	4.18	1.78			
^b^Efficacy expectations	710	4.82	1.87			
^c^Local environmental quality (last 3 months)	736	2.96	0.68			
Age	763	50.71	12.62			
**Subjective wellbeing for different age groups**						
< 30	64	6.48	2.26	61	6.55	2.14
30–60	509	6.76	2.32	505	6.93	2.18
>60	190	6.90	2.32	190	6.92	2.44
**Education**						
Did not complete school	4	0.5%				
Basic secondary school qualification	270	35.4%				
Secondary school diploma	235	30.8%				
Qualification for higher education	99	12.9%				
Graduation of University/University of applied sciences	155	20.3%				
**Income**						
Under 900	52	6.8%				
900–1.300 €	56	8.5%				
1.301–1.500 €	45	5.9%				
1.501–2.000 €	75	9.8%				
2.001–2.600 €	93	12.2%				
2.601–4.000 €	191	25.0%				
Above 4.000 €	242	31.7%				

a Scale ranging from 0 (extremely unhappy/extremely dissatisfied) to 10 (extremely happy/extremely satisfied).

b 7-point Likert scale ranging from 1 (strongly disagree) to 7 (strongly agree).

c Scale ranging from 1 (very bad) to 4 (very good).

It is important to keep in mind that data for both surveys were collected during summer months (June/July 2020 and June 2021). These were times in which the COVID-19 pandemic was less present in Germany than compared to wintertime, due to lower infection rates and hence fewer restrictions. Moreover, June 2021 presented a different situation than June 2020 since there were already vaccines available.

### Predictors of subjective wellbeing in June 2020 and June 2021

The five variables assessed in June 2020 explained about 10% of the variance in subjective wellbeing in June 2020 and 13% of the variance in June 2021 (Table 2). The more the COVID-19 pandemic was perceived as a threat (threat appraisal at T0), the lower was participants' subjective wellbeing in June 2020 and in June 2021. A higher perception of COVID-19 as a challenge/opportunity for change (challenge appraisal at T0) and higher efficacy expectations (T0) in June 2020 significantly predicted higher subjective wellbeing at both measurement times. Moreover, a more positive perception of the local environmental quality (T0) in June 2020 predicted a higher subjective wellbeing at both measurement times. In addition, a higher education significantly predicted a higher subjective wellbeing at both measurement times.

**Table 2:** Multiple moderator analysis predicting subjective wellbeing.

	**Difference in subjective wellbeing 2021 T1–2020 T0**				**Subjective wellbeing 2021 T0**				**Subjective wellbeing 2021 T1**			
	***b***	***SE***	β	***p***	***b***	***SE***	β	***p***	***b***	***SE***	β	***p***
Threat appraisal T0	−0.13	0.05	−.10	.008^**^	−0.19	0.06	−.13	.001^**^	−0.32	0.06	−.23	< .001^***^
Challenge appraisal T0	0.14	0.05	.12	.005^**^	0.10	0.06	.08	.078	0.24	0.06	.20	< .001^***^
Efficacy expectations T0	−0.04	0.05	−.03	.406	0.24	0.06	.19	< .001^***^	0.20	0.06	.16	< .001^***^
Local environ. quality T0	−0.10	0.11	−.03	.342	0.60	0.12	.18	< .001^***^	0.50	0.12	.16	< .001^***^
Education T0	−0.01	0.05	−.00	.831	0.20	0.05	.14	< .001^***^	0.19	0.05	.14	< .001^***^
p				.024				< .001				< .001
N	685											
R^2^	.02				.10				.13			

Displayed is the moderation analysis explaining the difference in subjective wellbeing between June 2020 and June 2021.

* *p* < .05.

** *p* < .01.

*** *p* < .001.

### Moderators of change in subjective wellbeing between June 2020 and June 2021 within participants

Participants perceiving the COVID-19 pandemic as a stronger threat to their health in June 2020 reported a lower subjective wellbeing over time (between June 2020 and June 2021), compared to those feeling less threatened (Table 2). This result suggests that the more participants perceived the COVID-19 pandemic as a threat to their health, the more they seemed to struggle with maintaining their subjective wellbeing over time compared to people with a lower threat perception. Accordingly, participants who perceived the COVID-19 pandemic more as a challenge and opportunity for change in June 2020 were more likely to show an increase in subjective wellbeing from June 2020 to June 2021.

We additionally explored potential differences in predictions of subjective wellbeing between younger (< 30 years) and older (>60 years) participants (Table 3). We chose this age range because for people older than 60 years, the risk of a severe corona disease progression increased and they were seen as a vulnerable group, particularly compared to younger people (< 30 years). For younger participants, most predictors failed to explain variance in their subjective wellbeing in a statistically significant way. However, results suggest that young participants' challenge appraisal in June 2020 was associated with their subjective wellbeing in June 2021. For older participants, a higher threat appraisal in June 2020 significantly predicted a lower subjective wellbeing in June 2020 and June 2021. However, the respective sample sizes were relatively small, and results should be interpreted cautiously.

**Table 3:** Multiple moderator analysis predicting subjective wellbeing.

	<**30 years old**									<**60 years old**								
	**Difference in subjective wellbeing 2021** ***T1***−**2020 T0**			**Subjective wellbeing 2020 T0**			**Subjective wellbeing 2021 T1**			**Difference in subjective wellbeing 2021 T1–2020 T0**			**Subjective wellbeing 2020 T0**			**Subjective wellbeing 2020 T1**		
	***b***	***SE***	***p***	***b***	***SE***	***p***	***b***	***SE***	***P***	***b***	***SE***	***p***	***b***	***SE***	***p***	***b***	***SE***	***p***
Threat appraisal T0	−0.42	0.24	.089	0.23	0.28	.401	−0.19	0.25	.464	0.01	0.08	.942	−0.44	0.10	< .001^***^	−0.43	0.11	< .001^***^
Challenge appraisal T0	0.45	0.18	.016^*^	0.03	0.20	.872	0.48	0.19	.013^*^	0.04	0.09	.648	−0.01	0.11	.946	0.03	0.12	.777
Efficacy expect. T0	−0.12	0.20	.536	0.23	0.22	.300	0.11	0.20	.587	0.03	0.09	.773	0.15	0.11	.177	0.17	0.12	.138
Local environ. quality T0	−0.64	0.40	.119	0.38	0.46	.421	−0.27	0.42	.526	0.01	0.20	.976	0.47	0.24	.054	0.48	0.26	.068
Education T0	−0.15	0.24	.550	0.20	0.28	.475	0.05	0.26	.835	0.07	0.12	.557	0.27	0.14	.058	0.34	0.15	.027^*^
*p*			*.131*			*.337*			*.068*			*.966*			* < .001*			* < .001*
*N*	57									174								
*R^2^*	.15			.10			.18			.01			.15			.14		

Results contrast younger (< 30 years) and older age groups (>60 years). Displayed is the moderation analysis explaining the difference in subjective wellbeing between June 2020 and June 2021 for different age groups.

* *p* < .05.

** *p* < .01.

*** *p* < .001.

## Discussion

The present study investigated how different evaluations of the COVID-19 pandemic and environmental factors affected Germans' subjective wellbeing during the pandemic. This study's approach is new in that we investigated the influences of the perception of and coping mechanisms within the pandemic on participants' subjective wellbeing over time while also considering the role of the perceived local environmental quality. Our results indicate that people are able to activate a range of positive coping mechanisms to maintain their subjective wellbeing in stressful situations extended in time, such as feelings of confidence (challenge appraisal) or collective efficacy expectations.

The results presented are in line with predictions from established psychological theories (14) and empirical evidence (10) and show that people can activate a range of coping mechanisms to deal with massive threats in order to maintain their subjective wellbeing even in times of crises. Besides these internal coping mechanisms, positive perceptions of the local environmental quality were also predictive of a higher subjective wellbeing in times of the pandemic, as could be shown for other countries as well (41). This suggests that, in times of crisis, people can regenerate their wellbeing in their local environments, e.g., by spending time outside in gardens or public green spaces (42). From this perspective, crises that impair the environmental quality such as the climate crisis appear even more threatening.

Our results on positive effects of peoples' efficacy expectation on their subjective wellbeing during the pandemic go in line with previous research's findings (22). These findings indicate that peoples' sense of belonging, pride, social actualization, and social integration that people develop in stressful situations help to decrease perceived stress in times of COVID-19. However, our results also indicate that individuals who perceived the COVID-19 pandemic as a stronger threat (threat appraisal) reported a lower subjective wellbeing. This result again is consistent with studies from different countries as shown by a meta-analysis on fear of COVID-19 (9). Moreover, lower education was predictive of a lower wellbeing. This suggests that individuals who tend to worry more and have lower socioeconomic resources (lower education) at their disposal struggle more to maintain their subjective wellbeing in crises.

In line with other surveys (5, 32), changes in subjective wellbeing from June 2020 to June 2021 were small and, in our case, not statistically significant. This suggests that the COVID-19 pandemic did not result in a general decrease of subjective wellbeing. As implied by the results of Mækelæ et al. (22) and a meta-analysis on mental health in the pandemic (43), peoples' increasing ability to manage their daily life under COVID-19 restrictions over time could result in a stabilization of their subjective wellbeing. On an intraindividual within-participants level, threat and challenge appraisal seemed to be relevant for changes in subjective wellbeing. Participants appraising the COVID-19 pandemic (2020) more as a challenge were more likely to maintain or even increase their subjective wellbeing from 2020 to 2021 compared to participants reporting lower levels of confidence or hope in 2020. Moreover, those expressing more anticipatory threat emotions such as feeling worried, fearful, and anxious in June 2020 were more likely to display a decreasing subjective wellbeing from 2020 to 2021. These results go in line with a recent study from Norway (44), suggesting that the way people adapt to the pandemic in its very beginning (health-promoting or impairing direction) has a crucial impact for their overall subjective wellbeing over time.

### Limitations

We are aware that characteristics of the COVID-19 pandemic change rapidly (e.g., the emergence of vaccines). This paper is built upon lines of thoughts we developed in spring 2020. However, we still incorporated studies and results that were published in the meantime. Overall, the studied predictors explained on average only about 15% of variance in participants' subjective wellbeing (June 2020 and June 2021). Their subjective wellbeing was therefore additionally influenced by other variables that we failed to measure. Other studies indicate that relevant psychological variables might be acceptance, gratitude, or optimism (45). Moreover, we did not measure objective risk factors for subjective wellbeing, such as medical conditions and illnesses. We measured participants' subjective wellbeing only twice, in June 2020 and June 2021. Given the frequent ups and downs of wellbeing in the pandemic, more frequent measures would have been informative to explain small variations in wellbeing. Furthermore, it is to be noted that we did not use clinical measures for subjective wellbeing. We decided to use a measure that is widely used as a short measure of subjective wellbeing and therefore easily comparable (European Social Survey). Future studies should research designs to identify groups that already suffer from an impaired wellbeing and investigate their specific abilities to cope with global threats and crises. Additionally, greatly differing conditions in different countries or populations make the generalization of the results difficult, both in regard to the severity with which the COVID-19 pandemic hit as well as the degree of restrictions that people were facing. However, several studies have pointed toward similar results in other countries as well [e.g., Norway (44), India or Turkey (9)].

## Conclusion

When transferring our results to the prediction of subjective wellbeing in other global threats such as the climate crisis, it seems key to support people in activating positive feelings of confidence, i.e., by focusing on joint mitigation actions, instead of focusing on incapacity. Some people, especially those worrying more about the infection and people with lower education, seem to struggle more than others with maintaining their subjective wellbeing during the COVID-19 pandemic. In other crises, there will be other groups most vulnerable to impairment of their wellbeing. Future research is needed to identify factors that support these vulnerable groups and foster their agency while maintaining or even raising their subjective wellbeing.

## Data availability statement

The raw data supporting the conclusions of this article will be made available by the authors, without undue reservation.

## Ethics statement

Ethical review and approval was not required for the study on human participants in accordance with the local legislation and institutional requirements. The patients/participants provided their written informed consent to participate in this study.

## Author contributions

HW: conceptualization, methodology, formal analysis, investigation, data curation, writing—original draft, and funding acquisition. VH and TS: investigation, writing—review and editing, and project administration. EM: conceptualization, investigation, resources, writing—review and editing, and funding acquisition. KS: conceptualization, investigation, writing—review and editing, and funding acquisition. All authors contributed to the article and approved the submitted version.
